# Effects of drug-resistant mutations on the dynamic properties of HIV-1 protease and inhibition by Amprenavir and Darunavir

**DOI:** 10.1038/srep10517

**Published:** 2015-05-27

**Authors:** Yuqi Yu, Jinan Wang, Qiang Shao, Jiye Shi, Weiliang Zhu

**Affiliations:** 1 Drug Discovery and Design Center, ACS Key Laboratory of Receptor Research, Shanghai Institute of Materia Medica, Chinese Academy of Sciences, 555 Zuchongzhi Road, Shanghai, 201203, China; 2 UCB Pharma, 216 Bath Road, Slough SL1 4EN, United Kingdom

## Abstract

Molecular dynamics simulations are performed to investigate the dynamic properties of wild-type HIV-1 protease and its two multi-drug-resistant variants (Flap + (L10I/G48V/I54V/V82A) and Act (V82T/I84V)) as well as their binding with APV and DRV inhibitors. The hydrophobic interactions between flap and 80 s (80’s) loop residues (mainly I50-I84’ and I50’-I84) play an important role in maintaining the closed conformation of HIV-1 protease. The double mutation in Act variant weakens the hydrophobic interactions, leading to the transition from closed to semi-open conformation of apo Act. APV or DRV binds with HIV-1 protease via both hydrophobic and hydrogen bonding interactions. The hydrophobic interactions from the inhibitor is aimed to the residues of I50 (I50’), I84 (I84’), and V82 (V82’) which create hydrophobic core clusters to further stabilize the closed conformation of flaps, and the hydrogen bonding interactions are mainly focused with the active site of HIV-1 protease. The combined change in the two kinds of protease-inhibitor interactions is correlated with the observed resistance mutations. The present study sheds light on the microscopic mechanism underlying the mutation effects on the dynamics of HIV-1 protease and the inhibition by APV and DRV, providing useful information to the design of more potent and effective HIV-1 protease inhibitors.

While human immunodeficiency virus (HIV) enters target cell, its RNA is transcribed into DNA through reverse transcriptase which then integrates into target cell’s DNA and rapidly amplifies along with the replication of target cell. The HIV-1 protease (HIV-1 PR) is essential to the replication and invasion of HIV as protease is responsible for cleaving large polyprotein precursors gag and releasing small structural proteins to help the assembly of infectious viral particles^1^,^2^,^3^. HIV-1 PR is a symmetrically assembled homo-dimer, consisting of six structural segments (Fig. 1a): flap (residues 43–58/43’–58’), flap elbow (residues 35-42/35’-42’), fulcrum (residues 11–22/11’–22’), cantilever (residues 59–75/59’–75’), interface (residues 1-5/1’-5’, 95-99/95’-99’), and active site (residues 23–30/23’–30’)^4^,^5^. So far two distinct conformations have been experimentally observed, mainly on the flap regions (two β-hairpins covering the large substrate-binding cavity): the flaps take a downward conformation towards the active site (closed state) when a substrate is bound, which, however, shift to a semi-open state when there is no bound substrate. The orientation of two β-hairpin flaps in the two states is reversed^6^,^7^.

Although no fully open state has been measured by X-ray crystallography experiment yet^3^,^8^,^9^,^10^, which is probably attributed to its short transient lifetime, reasonable speculation has been proposed that flaps could fully open to provide access for the substrate and then the residues of Asp25 and protonated Asp25’ in the active site of the protease aid a lytic water to hydrolyze the peptide bond of substrate, producing smaller infectious protein^11^,^12^. Subnanosecond timescale NMR experiment by Torchia and coworkers^13^,^14^,^15^ suggested that for substrate-free (apo) HIV-1 PR, the semi-open conformation accounts for a major fraction of the equilibrium conformational ensemble in aqueous solution, and a structural fluctuation is measurable on flap tips which is in a slow equilibrium (∼100 μs) from semi-open to fully open form. However, due to high flexibility of HIV-1 PR in aqueous solution, it is still difficult for NMR to provide detailed structural data for fully open conformation.

Molecular dynamics (MD) simulation, as an attractive alternative approach, has been extensively utilized to explore atomic-level dynamic information of flap motion. Scott and Schiffer^16^ reported irreversible flap opening transition in a MD simulation starting from the semi-open conformation of apo HIV-1 PR, which pointed out that the curling of flap tips buries the initially solvent accessible hydrophobic cluster and stabilizes the open conformation of HIV-1 PR. Similar but reversible flap opening event was also discovered by Tozzini and McCammon using coarse-grained model for 10 μs simulation^17^. In addition, the MD simulation by Hornak *et al.*^7^ captured rare event of semi-open conformation transiting to fully open conformation. These simulation studies showed that substrate-bound HIV-1 PR mainly takes closed conformation but transforms to semi-open conformation when substrate is removed. Therefore, both experiments and theoretical studies revealed the dynamic equilibrium among three conformations of HIV-1 PR (closed, semi-open, and fully open conformations), with the semi-open or closed conformation predominantly populated in apo or substrate-bound state, respectively^6^,^18^. It is worth noting that the open conformations found in the abovementioned MD simulations are somehow not exactly the same: although the active site is fully exposed, the flap tips are completely upward-oriented in the discovery of Hornak *et al.*^7^ but have downward curling conformation in the discovery of Scott and Schiffer^16^.

So far ten protease inhibitors have been approved by Food and Drug Administration (FDA), including squinavir (SQV), ritonavir (RTV), lopnavir (LPV), atazanavir (ATV), nelfinavir (NFV), indinavir (IDV), tipranavir (TPV), amprenavir (APV), fosamprenavir (FPV, prodrug of amprenavir), and darunavir (DRV or named as TMC114)^19^,^20^,^21^,^22^. These inhibitors bind to the active site of HIV-1 PR, block gag-pol, and thereby prevent the formation of mature virus particles *in vitro*. Nevertheless, because of the error-prone property of HIV-1 reverse transcriptase (HIV-1 RT), mutations in HIV-1 PR arise during the course of treatment, inducing drug-resistance to abovementioned inhibitors^4^. The flexibility of flap regions which is vulnerable to mutation controls the equilibrium among the three functional conformations: mutations in HIV-1 PR can make the flaps more flexible and destabilize closed conformation, which might increase the opening rate of flaps and thus release the inhibitor. Kinetic experiments show that the L90M, G48V, and L90M/G48V variants could reduce the binding affinity of inhibitors, which is caused by an increase in dissociation rates^4^. Perryman *et al.* reported that the protease variant with mutation sites in 80 s loops (V82F/I84V) shows more frequent and rapid flap curling than wild-type (WT) HIV-1 PR does^4^,^23^. Similarly, the I50V mutation in flap regions selected by APV^1^ shows more flexible flaps^24^, and single mutation distant from flap regions such as L63P or L10I can increase the flexibility of flap regions as well^25^. Hence, the dynamics of flaps changed by local or distal mutation is likely involved in increasing dissociation rates and thus reducing the efficiency of drugs^4^. More severely, the accumulation of single mutations causes serious cross drug resistance which reduces the potency of two most effective drugs, APV and the chemically similar inhibitor DRV (the single-ringed tetrahydrofuran (THF) group of APV is replaced by double-ringed bis-THF in DRV)^26^,^27^. Therefore, not only single mutation effect but also the cooperative effect of multi-mutations on HIV-1 PR should be studied to aid the design of novel drugs with better potency.

The isothermal titration calorimetry (ITC) experiment by King *et al.* indicated that the small structure difference of APV and DRV inhibitors lead to apparently different binding affinities towards WT HIV-1 PR and its two multi-drug-resistant (MDR) variants, namely Flap+ (L10I/G48V/I54V/V82A) and Act (V82T/I84V)^27^. Flap+has a combination of mutations mainly in the flap region whereas the mutations in Act are solely focused in 80 s (80’s) loop (Fig. 1). Accordingly, in comparison to WT protease, the binding affinity of the inhibitors to Flap+and particularly Act variants is reduced.

To understand the molecular mechanism underlying different binding affinity of the two chemically similar inhibitors to HIV-1 PR and the detailed mutational effects on the dynamics of protease as well as protease-inhibitor interactions, the present study ran long-time MD simulations on apo, APV-bound, and DRV-bound WT HIV-1 PR and its Flap+and Act variants. In addition, the absolute binding free energies of APV and DRV to the three HIV-1 PRs were calculated using free energy perturbation (FEP) method, which are very consistent with the experimental data^27^. The all-atom MD simulations observe a conformational transition from initial closed structure to a semi-open structure only for Act variant but not for WT protease and Flap+variant when there is no inhibitor bound. The detailed analysis reveals the origin of the amplified structural flexibility of the Act variant: the hydrophobic interactions between flaps and 80 s (80’s) loop residues (mainly I50-I84’ and I50’-I84) along with the hydrophobic interactions between the two flaps play important roles in maintaining the closed conformation of HIV-1 PR; the double mutation of the 80 s (80’s) loop residues (V82T/I84V) weakens the hydrophobic interactions between flap and 80 s (80’s) loop regions and thus destabilize the closed conformation. APV or DRV inhibitor binds with HIV-1 PR via hydrophobic interaction as well as hydrogen bonding: the two phenyls and one isobutyl groups of the inhibitor collaborate with the hydrophobic residues of I50 (I50’), I84 (I84’) and V82 (V82’) to create hydrophobic core clusters to enhance the stability of the closed conformation of the flaps; meanwhile the inhibitor forms multiple hydrogen bonds with the active site of HIV-1 PR. The combined change in the two kinds of protease-inhibitor interactions is correlated with the observed resistance mutations.

## Results

### Comparison of the binding thermodynamics measured by FEP calculation and experiment

In principle, the accuracy of theoretical simulation largely depends on the molecular force field used. In the present study, AMBER FF03 force field was used to model protein and the general amber force field (GAFF) was assigned to model APV and DRV inhibitors, respectively. To indicate the efficiency of the present study in measuring the molecular interactions of HIV-1 PR and the two inhibitors, the protease-inhibitor binding free energies were calculated for all complex systems under study by FEP method^28^. One can see from Table 1 that the calculated value of the binding free energy is in well agreement with the experimental data for each complex system. In addition, for each inhibitor, its binding free energy with WT HIV-1 PR is more negative than those with Flap+and Act. Meanwhile, the binding free energy of DRV is generally more negative than that of APV with respect to individual proteases, suggesting that the force fields used here can essentially reflect the change in protease-inhibitor binding interactions induced by the residue mutation of HIV-1 PR as well as the structure change of inhibitor.

### Higher flap flexibility in Act variant than in wild-type HIV-1PR and Flap+variant

**Figure 2:**
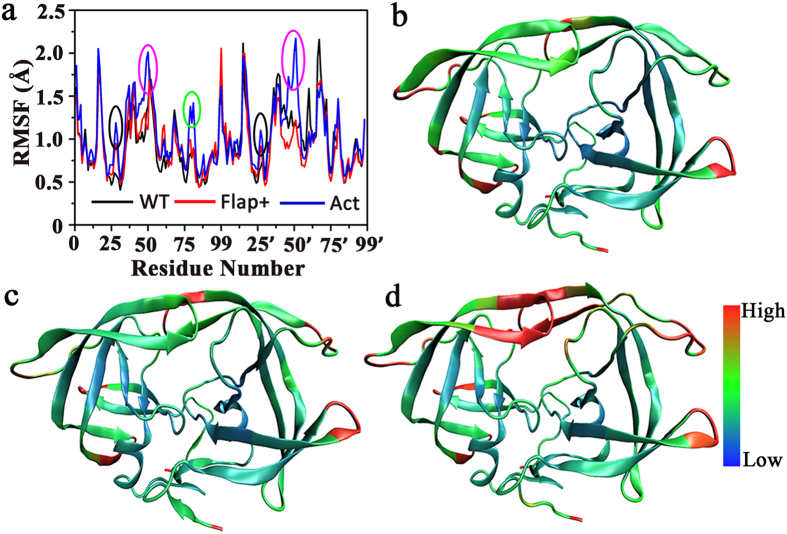
(**a**) Root-mean-square fluctuation (RMSF) *vs* residue index for apo WT HIV-1 PR and the variants of Flap+and Act (residues in active site, flap, and 80s loop are highlighted by black, pink, and green circles, respectively). (**b**–**d**) Average RMSF of residues in apo WT, Flap+, and Act proteases superposed onto the corresponding crystal structures, with the most (least) variable regions depicted in red (blue).

To see the effect of amino acid mutation on the structure stability of apo HIV-1 PR, root-mean-square fluctuations (RMSFs) of individual residues for the three apo proteases were calculated. As shown in Fig. 2a, the RMSFs in Flap+variant have similar shape but slightly smaller values than those in WT HIV-1 PR. Intriguingly, the RMSFs in Act variant have apparently larger values, particularly for residues in flap (50 s/50’s), 80 s loop and active site (25-30/25’-30’), suggesting larger flexibility in these regions (Fig. 2a–d).

**Figure 3:**
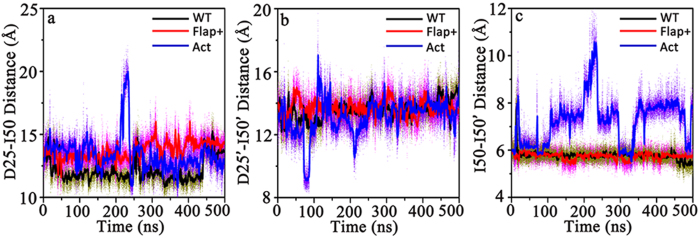
Time series of (**a**) D25-I50 C_α_ distance, (**b**) D25’-I50’ C_α_ distance, and (**c**) I50-I50’ C_α_ distance of the apo WT HIV-1 PR and its Flap+and Act variants.

**Figure 4:**
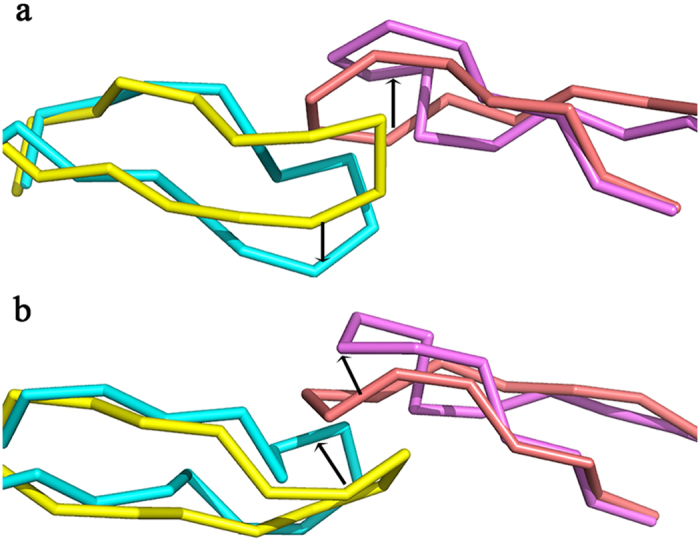
Comparison of the semi-open conformation of the flaps in Act variant observed in MD simulation to the closed conformation from (**a**) front view and (**b**) top view. The flaps in chains A and B in closed conformation are shown with pink and yellow ribbons and the corresponding flaps in semi-open conformation are shown with purple and cyan ribbons. Directions of flap motions are highlighted by black arrows.

To inspect the motion difference on flaps among the three apo systems, we calculated the inter-residue distances of I50-I50’, D25-I50, D25’-I50’ that have been often used to reflect the horizontal and vertical motion of flaps^4^,^24^,^29^. Figure 3 shows that there is a remarkable difference in the motion of flaps in Act as both WT and Flap+proteases show similar motion inferred from the mild fluctuation of the three abovementioned distances. One can find that at ~220 ns, the three inter-residue distances in Act variant reach their climaxes simultaneously, corresponding to the large motion of flaps at this time. Snapshot conformation was extracted to visualize the structure at 220 ns and a semi-open conformation was achieved. As shown in Fig. 4, incomparison to initial closed conformation of flaps, this semi-open structure has its flap in one monomer (chain A) moving upward while the flap in the other monomer (chain B) is slightly moving downward (Fig. 4a). Furthermore, the two flaps also have lateral motions (Fig. 4b), which is consistent with the observation in the simulation study of Tozzini and McCammon^17^.

A “curling” motion of flaps in the transition between closed and open states of HIV-1 PR was suggested by NMR experiment and multiple simulations^13^,^14^,^16^: The highly flexible flap tips are curling back into the protease and triggers the opening of flaps. TriCa (the angle between three adjacent Cα) of residues 48-49-50 (48’-49’-50’), which can be used as a reaction coordinate to illuminate the curling motion of flaps tips^4^,^24^, is plotted in Fig. S1 in the supplementary material online. The TriCa values corresponding to the curled-in and curled-out states are about 110° and 125°, respectively. One can see from Fig. S1 in the supplementary material online that the flap tip in chain A of Act variant stays in curled-in state in the simulation time range of 150 ~ 230 ns, consistent with the upward motion of the flap in the semi-open conformation since the curled-in of the flap tip triggers its opening. In contrast, the WT HIV-1 PR and Flap+variant show little or none curled-in behavior of the flap tips. Therefore, we can conclude that Act variant confers higher flexibility on the flap regions than the WT HIV-1 PR and Flap+variant, which promotes the opening of flaps in Act but not in the other two proteases.

To further assess the amplified dynamic properties of Act variant with respect to the WT and Flap+, the distance distributions involving the residues in flap tips were calculated for the three apo proteases (Fig. S2 in the supplementary material online). The distance between the tip of the flaps (50-50’) is centered at similar position for WT protease and Flap+but is shifted to larger value for Act. The flap tip in one monomer is farther away from the 80 s loop in the other monomer (80-50’) in Act in comparison to the WT and Flap+. In addition, the intra-monomeric C_α_ distance of 50-80 is shortest in Flap+, in the middle in WT, and longest in Act. The inter-monomeric distance between the flap tip and active site (25-50’) remains unchanged among the three protease systems but the intra-monomeric distance of 25-50 is elongated in Flap+and Act mutants. The changes in these distances further indicate the more open conformation of the flaps in Act than those in the other two proteases. In contrast to rather rigid active site region, the inter-monomeric distance of 80 s loops (80-80’) which form the inner walls of the active site are shortened in Flap+but is elongated in Act compared to that in WT protease. The features of all abovementioned residue-residue distance distributions in WT and Flap+are in well agreement with the previous simulation results for the same proteases by Cai *et al.* (see Fig. 5 in Ref. 38).

### Important roles of hydrophobic interactions between Ile50 (Ile50’) in flap and Ile84’ (Ile84) in 80’s (80 s) loop in adjusting the dynamics of flap

**Figure 5:**
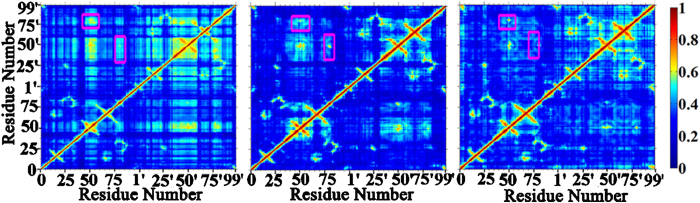
Residue-residue correlation in WT (left panel), Flap+(middle panel), and Act (right panel) HIV-1 PR. The correlation between the flap tip in chain A (B) and 80’s (80 s) loop is highlighted by pink square.

To explore the inherent reason why the flap flexibility is amplified in Act variant, general correlation analysis, developed by Oliver F. Lange^30^, was performed for the three apo protease systems under study to reveal the correlation between flaps and the remaining parts of HIV-1 PR (Fig. 5). The strongest correlation can be seen between the flap tips in chain A (residues around I50) and chain B (residues around I50’), which can be explained by the direct inter-residue interactions between the two flaps, e.g., the hydrophobic interactions of I47-I50’, I50-I54’ pairs and the counterparts of I47’-I50 and I50’-I54. In addition, the flap tip in chain A (B) also shows strong correlation with 80’s (80 s) loop in WT HIV-1 PR. This correlation is, however, attenuated in Flap+and particularly in Act variant.

**Figure 6:**
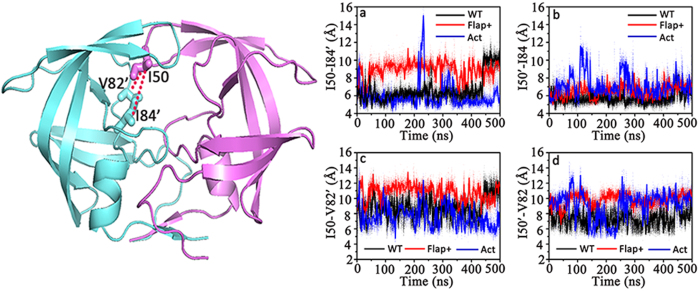
Time series of the side-chain distance of (**a**) I50-I84’, (**b**) I50’-I84, (**c**) I50-V82’, and (**d**) I50’-V82 of the apo WT HIV-1 PR and its Flap+and Act variants. Isoleucines in 84 and 84’ positions are replaced by valines in Act, valines in 82 and 82’ are replaced by alanines in Flap+and threonines in Act, respectively. The interactions of these hydrophobic residues in WT HIV-1 PR are shown in left panel.

Hydrophobic interactions between I50 in flap A and I84’ in 80’s loop as well as I50’ in flap B and I84 in 80 s loop are the key interactions between flap and 80 s (80’s) loop regions which can be clearly seen in the crystal closed structure of WT HIV-1 PR (Fig. 6). Such hydrophobic interactions along with the hydrophobic interactions between the two flaps might contribute for fastening the flaps. As the hydrophobic side-chain of I84 is shortened in Act (I84 → V84), its contribution should be certainly decreased. In order to analyze the hydrophobic interactions of I50-I84’ and I50’-I84, the side-chain distances of the two pairs are plotted in Fig. 6a for WT HIV-1 PR and the Flap+and Act variants. One can see that the side-chain distance of I50-I84’or I50’-I84 mainly stays in low region (~6.0 Å), indicating a strong hydrophobic interaction between these residues in WT HIV-1 PR. In Flap+, the distance of I50’-I84 still keeps steady and small but the distance of I50-I84’ is slightly increased, suggesting weaker hydrophobic interaction between flap and 80 s loop. In contrast, the replacement of I84 by valine leads to the large fluctuation of the side-chain distance of I50-V84’ in Act and a high peak can be seen clearly at ~220 ns, corresponding to the partial opening of the flaps (Fig. 3 and 4). In addition, the fluctuation of I50’-V84 side-chain distance in Act is also larger than those in the other two proteases (Fig. 6b). Peaks can be also seen at the position of 70 ~ 100 ns, corresponding to a slight upward motion of the flap in chain B (see Supplementary Fig. S3 online). These observations are consistent with our speculation that the hydrophobic interaction between I50 (I50’) in flap and I84’ (I84) in 80’s (80 s) loop plays an important role in locking the flaps in closed conformation and the mutation of I84 by less hydrophobic residue might loosen the flaps.

Another hydrophobic residue in 80 s (80’s) loop, V82 (V82’), could also form hydrophobic interaction with the flap residue I50’ (I50) but its strength is weaker than that of I50-I84’ (I50’-I84), as revealed by the longer distance in Fig. 6c. The replacement of V82 by alanine or threonine further elongates the inter-residue distance and thus further weakens the hydrophobic interactions. Therefore the interactions from V82 to I50’ and from V82’ to I50 are at least not as important as the interactions from I84 to I50’ and from I84’ to I50 in stabilizing the closed conformation of HIV-1 PR.

### Molecular interactions between HIV-1 PR and DRV/APV inhibitors

**Figure 7:**
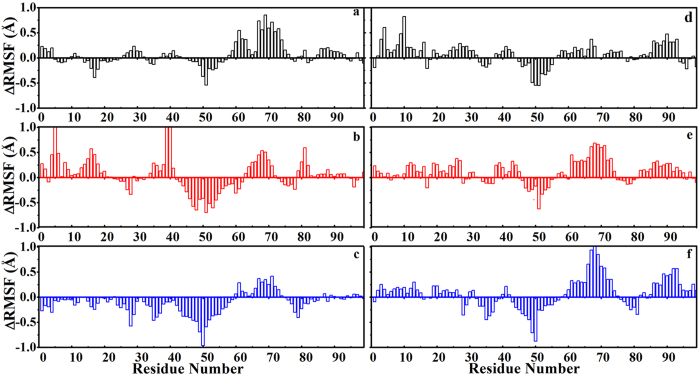
Change in RMSF fluctuations of C_α_ atoms in one monomer averaged from MD simulations between (**a**–**c**) APV-bound and apo, or (**d**–**f**) DRV-bound and apo WT HIV-1 PR (black) and Flap+(red) and Act (blue) variants, respectively.

To see the influence of inhibitor binding on the flap dynamics of HIV-1 PR, the distances between D25 and I50, D25’and I50’, I50 and I50’ for APV or DRV bound WT, Flap+, and Act proteases are plotted in Fig. S4 in the supplementary material online. The decrease in the fluctuation of these distances can be seen in the presence of these inhibitors, particularly for Act variant. In addition, the decrease in distance fluctuation is generally more apparent for the binding of DRV than the binding of APV, suggesting stronger inhibiting effects of the former inhibitor than the latter. The change in RMSF of the C_α_ atoms of individual residues in one monomer of HIV-1 PR induced by the inhibitor binding was also measured. One can see from Fig. 7 that the regions involved in the inhibitor contacting (flaps, active site, and 80 s loop) are less flexible (with negative ΔRMSF) as APV or DRV is bound. This restriction is more pronounced for Act variant. Interestingly, the other regions which are not subjected to the contacts from the inhibitor become more flexible in the inhibitor-bound state. These two effects compensate with each other and as a result the average flexibility of the protease might be not highly influenced by the inhibitor binding, consistent with the observation in previous molecular simulation by Cai *et al.*^31^.

**Figure 8:**
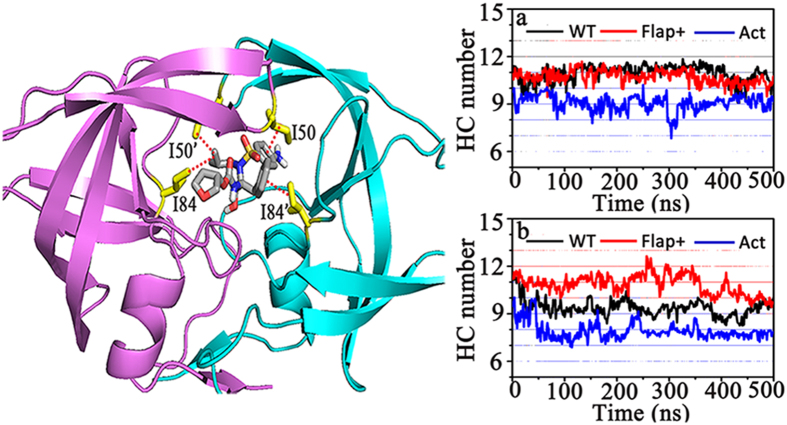
Time series of the hydrophobic contacts (HC) number between the HIV-1 PR and (**a**) APV or (**b**) DRV inhibitor. A representative structure of HIV-1 PR containing the hydrophobic interactions among the flap, 80 s (80’s) loop, and APV inhibitor is shown in left panel.

As discussed earlier, the hydrophobic interactions of I50-I84’ and I50’-I84 play an important role in holding the flaps in their closed conformations. As DRV or APV enters the binding pocket of HIV-1 PR, the positioning of the inhibitor allows its two phenyl groups and one isobutyl group to participate into the hydrophobic interactions with the hydrophobic pairs of I50-I84’ and I50’-I84, which creates stable hydrophobic core clusters and thus could enhance the stabilization of the closed conformation of HIV-1 PR (Fig. 8). The time series of the total number of hydrophobic contacts (HC) between APV or DRV and the protease was plotted in Fig. 8 for all complex systems. In addition, the total number of hydrophobic contacts was averaged throughout the simulation trajectory for each complex system and the error was estimated with 10 ns/block averaging. One can see that the numbers are more or less similar for WT HIV-1 PR and Flap+variant no matter which inhibitor is bound (the averaged values are 10.93 ± 0.34 for WT-APV, 10.71 ± 0.26 for Flap+-APV, 9.38 ± 0.34 for WT-DRV and 10.86 ± 0.49 for Flap+-DRV, respectively). The error bars are quite small compared to the detailed HC numbers, indicating the simulation convergence. In contrast, the hydrophobic contacts in Act are apparently less (the averaged values are 9.07 ± 0.23 for Act-APV and 7.91 ± 0.38 for Act-DRV), which can be attributed to the two mutations of V82 and I84 by less hydrophobic residues in the protease.

**Figure 9:**
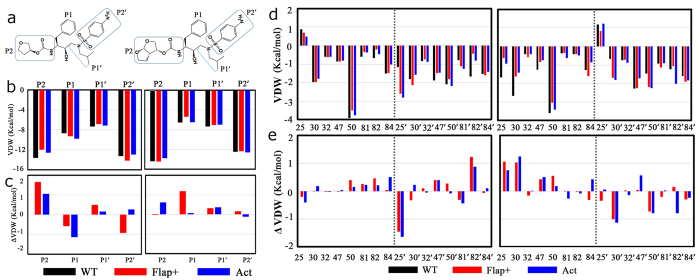
(**a**) The structures of APV and DRV with the inhibitor moieties P2-P2’ indicated. (**b**) vdW interaction energy of APV (left) or DRV (right) moieties for contacts with WT, Flap+, or Act protease active site in the equilibrium structures. (**c**) The changes in vdW interaction energy in mutant structures (Flap+and Act) relative to the WT complex (positive values indicate loss of contacts). (**d**) The vdW interaction energy of individual active site residues to APV (left) and DRV (right). (**e**) The changes in vdW interaction energy between individual active site residues and inhibitor in mutant structures (Flap+and Act) relative to the WT complex. Only the active site residues having considerable changes relative to WT are presented.

The van der Waals (vdW) contacts between specific APV or DRV moieties (Fig. 9a) and HIV-1 PR were analyzed in details (Fig. 9b). The calculated vdW interaction energies between various moieties of DRV and WT protease (Fig. 9b right) are more or less close to the counterparts as shown in Fig. 2B of Ref. 41. The mutation of WT protease to Flap+or Act leads to the loss of vdW interaction energies between the protease and DRV moieties. The detailed values of the loss of vdW interaction energies for all moieties of DRV induced by the Act mutation (0.06 kcal/mol for P1, 0.62 kcal/mol for P2, 0.36 kcal/mol for P1’, and -0.12 kcal/mol for P2′, see Fig. 9c right) are slightly smaller than the previously reported values (0.30 kcal/mol for P1, 0.20 kcal/mol for P2, 0.83 kcal/mol for P1’, and 1.11 kcal/mol for P2′ in Table 1 of Ref. 32). Therefore, while Ref. 32 indicated that the impact of Act mutation on DRV contacts is mainly focused on the P1’ and P2′ moieties, the present study suggests that the impair is mainly on P1′ and P2. The impact of Flap+mutation on DRV contacts is, however, larger at the central P1 and P1’ than the marginal P2 and P2’ moieties (Fig. 9c right). For APV inhibitor, the residue mutation induces more complex impact on protease-inhibitor contacts: the mutation of WT to Flap+impairs vdW interaction of P1’ and P2 moieties but meanwhile promotes vdW interaction of the remaining moieties of APV; the mutation of WT to Act impairs vdW interaction of the most moieties (P2, P1’, and P2′) but meanwhile promotes vdW interaction of the P1 moiety of APV.

The vdW interaction energies between the whole molecule of APV or DRV inhibitor and individual representative active site residues were also calculated and depicted in Fig. 9d, along with the corresponding changes in vdW interaction energy in mutant structures (Flap+and Act) relative to the WT complex (Fig. 9e). One can see that DRV induces asymmetry in protease-inhibitor contacts (Fig. 9e right). As a result, although the residue mutating is identical in both monomers, the effects of the mutations on protease-inhibitor contacts are not uniform in the two monomers. As a matter of fact, it is mainly the residues of 25, 30, 47, and 50 in one monomer that lose vdW interactions in DRV-bound flap+and Act variants. In contrast, the impairment of vdW interactions towards APV spreads over the two monomers in flap+and Act variants (Fig. 9e left).

**Figure 10:**
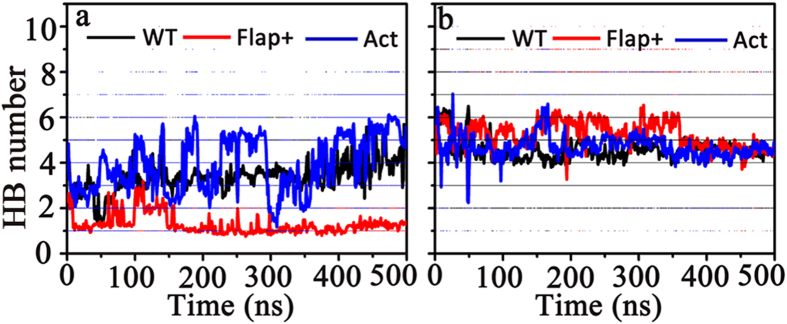
Hydrogen bond (HB) number formed between HIV-1 PR and (**a**) APV or (**b**) DRV.

Hydrogen bonding interaction along with the hydrophobic interaction are the main contributors in protease-inhibitor binding. The number of protease-inhibitor hydrogen bonds (HB) was calculated for the complex systems under study (Fig. 10). One can see that the HB numbers formed by DRV with WT protease and the variants are more or less similar (the averaged values are 4.58 ± 0.30 for WT-DRV, 5.27 ± 0.42 for Flap+-DRV, and 4.70 ± 0.25 for Act-DRV, respectively). In addition, the HB number between APV and WT protease (3.39 ± 0.41) is slightly smaller than that between DRV and WT protease. The HB number between APV and Act fluctuates a lot and the HB number between APV and Flap+is sharply decreased in comparison to those between DRV and corresponding protease systems. Therefore, while the average HB number between APV and Act is 3.92 ± 0.82, the average number between APV and Flap+is only 1.28 ± 0.32. Generally speaking, the electrostatic interactions from APV to HIV-1 PR are weaker than those from DRV. Considering the similar numbers of the protease-inhibitor hydrophobic contacts for APV and DRV (Fig. 8a,b), the less electrostatic interaction of APV to HIV-1 protease could be the main reason for the experimentally observed weaker binding of APV than DRV.

As a critical hydrogen bond, hydrogen bond formed between D25 and/or D25’ residue and the hydroxyl group of APV or DRV is given additional attention^22^. While D25’ is protonated in the present simulation, the hydroxyl group of either inhibitor mainly forms hydrogen bond with the side-chain OD2 atom of D25 (see Supplementary Fig. S5 online). The distance between side-chain OD2 atom of D25 and the hydroxyl oxygen (O3) of the inhibitor was calculated (see Supplementary Fig. S6 online). In WT HIV-1 PR, the hydrogen bond distance between D25 and APV mainly stays at low value (~2.7 Å) with occasional jumping to relatively high value (~4.0 Å). The hydrogen bonding of D25 and DRV is quite stable since its distance always keeps at low value. While the WT HIV-1 PR is mutated to Act or Flap+, the hydrogen bonding becomes less stable, as revealed by larger fluctuation in the hydrogen bonding distance between D25 and APV or DRV. Meanwhile, the THF of inhibitors can also form hydrogen bonds with surrounding protease residues (see Supplementary Fig. S7 online). For instance, the O6 atom of THF in APV could form two hydrogen bonds with the backbone amide hydrogens of D29 and D30 although the occupancies are low (13.63 ± 8.80% and 16.28 ± 9.82% for D29-APV and D30-APV in WT HIV-1 PR, 50.48 ± 13.30% and 61.18 ± 14.94% in Act but only 3.72 ± 1.30% and 0.05 ± 0.02% in Flap+). The O6 of bis-THF in DRV has enhanced hydrogen bonding ability with protease (59.37 ± 10.67% and 83.70 ± 8.29% for D29-DRV and D30-DRV in WT HIV-1 PR, 17.28 ± 5.22% and 16.47 ± 6.50% in Act, and 51.67 ± 8.07% and 77.18 ± 8.00% in Flap + ). In addition, the O7 in bis-THF of DRV can also form additional hydrogen bond with residue D29 (occupancy is 45.07 ± 12.72% in WT, 89.03 ± 3.76% in Act, and 66.47 ± 4.37% in Flap + HIV-1 PR). Therefore, more hydrogen bonds from DRV than APV can be formed with protease.

**Figure 11:**
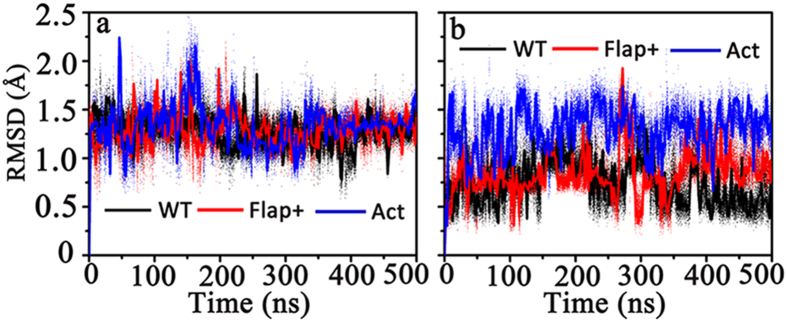
Time series of RMSD for (**a**) APV and (**b**) DRV with respect to their initial configurations in WT, Flap+and Act HIV-1 PR.

It has been reported that water may mediate the binding interaction of inhibitor and HIV-1 PR^20^,^34^,^33^. A highly conserved “bridge” water molecule^24^ connecting the flap region and inhibitor can be found in the crystal structure of HIV-1 PR: the oxygen atom of this bridging water forms two hydrogen bonds with the backbone amide hydrogens of I50 and I50’ and meanwhile the hydrogen atoms of the water form two hydrogen bonds with the O2 and O5 atoms of APV or DRV (see Supplementary Fig. S8 online). The occupancy of the bridging water is defined as the percentage of protein structures containing a water molecule connecting the flap region and inhibitor as described above once the thermal equilibrium has been reached (e.g., after 10 ns of the simulation), based on the entire simulation data. The occupancy can have values from 0 to 100% and such definition sketches a reasonable space scope for bridging water. In addition, since the bridging water can form four hydrogen bonds simultaneously with the flap region of protease as well as drug backbone which undoubtedly restricts the dynamics of the protease, the occupancy of bridging water could to some extent reflect the efficacy of drug. Table 2 indicates the average value of the bridging water occupancy as well as the average distance of hydrogen bonds with the bridging water involved. The significantly high values of the bridging water occupancy in WT-DRV and Flap+-DRV complexes imply that the two complex systems contain bridging water consistently. However, when DRV is bound to Act or APV is bound to any HIV-1 PR system, the conservation of the bridging water is largely decreased. To understand why the bridging water is more conserved in DRV-bound protease systems, we next calculated the RMSD values of the two inhibitors with respect to their initial configurations. Figure 11 shows that the RMSD value of DRV is relatively lower than that of APV in each protease system. In addition, the RMSD fluctuation of either APV or DRV is largest as it is bound with Act protease. These tendencies are consistent with the occupancy of the bridging water (Table 2), suggesting that the stability of the hydrogen bond network of the bridging water is mainly dependent with the configuration stability of the inhibitor.

## Discussion

The appearance of drug-resistant HIV-1 PR mutations becomes one of the main challenges for AIDS therapy. Understanding the molecular mechanism of drug resistance is critical to the design of new drugs, which requires comprehensive information of the binding interactions of inhibitors and their influence to the dynamics of WT HIV-1 PR and the drug-resistant variants. The binding of APV with HIV-1 PR is tighter than the first-generation inhibitors by ~1 order of magnitude^35^. The chemically similar inhibitor, DRV, which has a second THF ring, binds with the protease even more tightly than APV by 2 orders of magnitude. Through comparing the crystal structures of APV and DRV bound WT HIV-1 PR and its MDR variant (L63P, V82T, and I84V) and calculating their corresponding binding thermodynamics, King *et al.* observed that the binding of the two inhibitors to the MDR variant is impaired but the impaired binding is still more favorable than those of first-generation inhibitors^36^. It was found that the I84V substitution in HIV-1 PR reduces van der Waals (vdW) interactions with the inhibitors, which might account for the reduced binding affinities of both APV and DRV. The potency of APV and especially DRV against MDR viruses was attributed by the authors to a combination of their high binding affinity and close fit with the binding pocket^36^. More recent ITC experiment of a series of inhibitors including APV and DRV binding to WT HIV-1 PR and two variants (Flap+and Act) by the same research group indicated that the Flap+variant exhibits extremely large enthalpy-entropy compensation for all inhibitors, suggesting that the drug-resistant mutations in Flap+directly modulate the binding thermodynamics of inhibitors^27^. The molecular mechanics Poisson-Boltzmann (or Generalized Born) surface area (MM-PB/GBSA) and thermodynamic integration (TI) calculation of the binding free energy of DRV towards the WT HIV-1 PR and its Flap+and Act variants indicated that the vdW interaction energy is dominant whereas the contribution of electrostatic interaction energy is minor in the total binding free energy of DRV to protease^37^. The crystal structure comparison of flap variants (I50V, I54V, and I54M) bound with SQV and variants (G48V, I54V, and I54M) bound with DRV suggested that the change in polar interactions between protease and inhibitors have the best correlation with observed resistance mutations^40^.

In the present study, the dynamic properties of WT HIV-1 PR and its two variants Flap+and Act as well as their interactions with APV and DRV inhibitors were investigated with all-atom MD simulations and FEP calculation. The binding free energies between the inhibitors and proteases achieved by FEP calculation are in well agreement with the experimental data, suggesting that the molecular force fields used here are suitable for the description of the protease-inhibitor interactions. Using the same force fields, the comparative MD simulations obtained results (e.g., the change in protease RMSF induced by inhibitor binding and mutational effects, the change in vdW interaction energy between inhibitor moieties and protease induced by mutational effects, and the distribution of residue-residue distance) comparable to multiple previous MD simulations^31^,^32^,^38^,^41^. Higher structural flexibility of Act variant is observed in comparison with WT protease and Flap+variant. Specifically, starting from the closed conformation, a semi-open conformation is reachable for Act but not WT and Flap+proteases when there is no inhibitor bound. The detailed analysis indicates that besides the hydrophobic interactions between the two flaps, the hydrophobic interactions between flaps and 80 s (80’s) loop residues (mainly I50-I84’ and I50’-I84) also play important roles in maintaining the closed conformation of HIV-1 PR. As the hydrophobic side-chain of I84 is shortened (I84 → V84) and V82 is substituted by hydrophilic residue (V82 → T82) in Act, the contribution of the hydrophobic interaction from 80 s loop is certainly decreased, leading to the amplified structural flexibility of flaps. The essential role of the hydrophobic core including I50 (I50’), I84 (I84’), and V82 (V82’) in modulating the activity of HIV-1 protease was also emphasized by Mittal *et al.* in their recent site-directed cysteine cross-linking experiment^39^.

The interactions between HIV-1 PR and APV or DRV inhibitor are also illuminated in the present study. Both hydrophobic and hydrogen bonding interactions contribute to the protease-inhibitor binding. For instance, the two phenyl groups and one isobutyl group of APV or DRV participate into the hydrophobic interactions with the hydrophobic pairs of I50-I84’ and I50’-I84 of HIV1- PR, which creates stable hydrophobic core clusters among the flap, 80 s (80’s) loop, and the inhibitor and thus could enhance the stabilization of the closed conformation of HIV-1 PR. The double mutation of the two hydrophobic residues by less hydrophobic ones in Act reduces the total number of hydrophobic contacts. As a result, although the flexibility of the HIV-1 PR structure is constrained by the binding of APV or DRV, the constraint in the structural flexibility of Act is the lowest. On the other hand, either APV or DRV can also form hydrogen bonds with the active site of HIV-1 PR, e.g., the hydrogen bindings of the hydroxyl group of the inhibitor to D25 and the oxygen in THF group of the inhibitor to D29 and/or D30 of HIV-1 PR. The additional THF ring of DRV allows it to form additional hydrogen bond with HIV-1 PR in comparison with APV. The inhibitors can also connect to the flap region via the hydrogen binding of a bridging water. The presence of fewer hydrogen bonds from APV to HIV-1 PR, which might be correlated with the less conformational stability of APV in the binding pocket, accounts for the experimentally observed weaker binding of APV than DRV to HIV-1 PR. As a matter of fact, a novel inhibitor with tris-THF, GRL-0519 that is under clinical trial, demonstrates better potency than DRV, for the more complicated hydrogen bond network between tris-THF and the active site^42^. In summary, the present study reveals the microscopic mechanism of mutational effects on the dynamic properties of HIV-1 PR and provides an atomic-level picture of the binding interactions between APV/DRV inhibitors and HIV-1 PR as well as the structure-affinity relationship. We anticipate that the present study could provide useful information to the future design of more potent and effective HIV-1 PR inhibitors.

## Methods

### Molecular dynamics simulation

The initial coordinates of APV-WT, APV-Flap+, APV-Act, DRV-WT, DRV-Flap+, and DRV-Act complexes were obtained from the protein data bank (PDB) and their PDB codes are 3EKV, 3EKP, 1T7J, 1T3R, 3EKT, and 1T7I, respectively^27^,^43^. The corresponding apo HIV-1 PR systems were obtained by removing the inhibitors from corresponding inhibitor-bound complex systems. All the crystal water molecules were retained. Considering the importance of the protonation of Asp25/Asp25’ in the HIV-1 PR, a proton was added to the oxygen atom OD2 in Asp25’ in chain B of HIV-1 PR and kept undetached in the simulation^24^,^44^. All simulations were carried out by the program GROMACS version 4.5.3 using the NPT ensemble and periodic boundary condition. The AMBER FF03 force field^45^ was applied to model protein and the general amber force field (GAFF)^46^ was assigned to model the two inhibitors (APV and DRV). In each simulation, the apo or inhibitor-bound protease was solvated in a cubic box with TIP3P water molecules^47^, keeping the boundary of the box at least 14 Å away from any protein atoms. Counterions were added for charge neutralization of whole simulation system. Each simulation system was first subjected to energy minimization using the steepest descents algorithm. Subsequently, a 5 ns MD simulation was carried out to heat the system to 300 K with the protein and the inhibitor fixed using a harmonic restraint (force constant = 10 kcal/mol/Å^2^), followed by another 5 ns MD simulation with the protein C_α_ atoms and inhibitor fixed. Finally, based on the relaxed system, the long-time equilibrium simulation (production run) was run without any constraints for 500 ns. The temperature of each system was maintained at 300 K using a novel V-rescale thermostat^48^ with a response time of 1.0 ps. The pressure was kept at 1 bar using the Parrinello-Rahman pressure coupling scheme^49^ ( τ = 1 ps). The cutoff for Lennard-Jones interactions was set as 12 Å and the electrostatic interactions were calculated using the particle Mesh Ewald (PME) algorithm^50^ with a real-space cutoff of 12 Å. The LINCS^51^ method was used to restrain bond lengths that including hydrogen atoms, allowing an integration step of 2 fs.

### Free energy perturbation calculation

The binding free energies of the inhibitors (APV and DRV) to HIV-1 PR and its Flap+and Act variants were calculated using the free energy perturbation method described by Shirts *et al*^28^., as combinations of free energies of mutation of inhibitor to vacancy at the protein binding site and in bulk water. Simulations were performed separately at 21 different alchemical intermediate λ values: 0, 0.05, 0.10, 0.15, 0.20, 0.25, 0.30, 0.35, 0.40, 0.45, 0.50, 0.55, 0.60, 0.65, 0.70, 0.75, 0.80, 0.85, 0.90, 0.95 and 1. In these simulations, the coupled state (λ = 1) corresponds to a simulation where the solute (APV or DRV) is fully interacting with the environment and the uncoupled state (λ = 0) corresponds to a simulation where the solute does not interact with the environment. Each window corresponds to an independent simulation that includes 1.5 ns of equilibration and subsequent 3.5 ns of data collection. The free energies were computed using the Bennett acceptance ratio (BAR)^52^. The simulation temperature was kept constant at 300 K by coupling the system to a Nose´-Hoover thermostat^53^,^54^ (τ = 0.5 ps) and the pressure was kept at 1 bar using the Parrinello-Rahman pressure coupling scheme (τ = 1 ps). The cutoff for Lennard-Jones interaction was set as 12 Å.

### Generalized cross-correlation analysis

Cross-correlations of residues in simulation systems were calculated based on mutual information between all C_α_ atoms of protein using the generalized correlation analysis approach developed by Lange and Grubmüller^30^. The *g_correlation* module in the GROMACS package was applied for the analysis.

## Additional Information

**How to cite this article**: Yu, Y. *et al.* Effects of drug-resistant mutations on the dynamic properties of HIV-1 protease and inhibition by Amprenavir and Darunavir. *Sci. Rep.* **5**, 10517; doi: 10.1038/srep10517 (2015).

## Supplementary Material

Supplementary Information

## Figures and Tables

**Figure 1:**
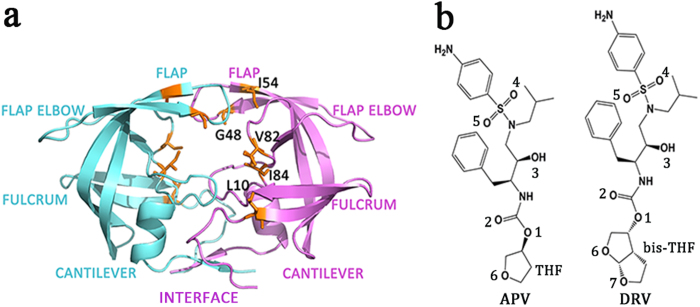
(**a**) HIV-1 protease structure (PDB code: 1T3R) in inhibitor-bound state. HIV-1 protease is shown in purple and cyan colored cartoons for chain A and chain B, respectively. Mutation sites (L10, G48, I54, V82, and I84) are shown in orange colored licorice representation. (**b**) Structures of APV and DRV inhibitors (key oxygen atoms involved in the protease-inhibitor interactions are labeled with numbers).

**Table 1:** Binding free energies of inhibitors to WT HIV-1 PR and its Flap+and Act variants and the comparison to experimental data.

		**WT**	**Flap+**	**Act**
APV	Calc.	−13.51 ± 0.98	−11.65 ± 0.59	−10.36 ± 0.96
	Expt.	−12.4 ± 0.3	−11.7 ± 0.0	−11.4 ± 0.2
DRV	Calc.	−16.01 ± 0.79	−14.27 ± 0.57	−11.93 ± 0.67
	Expt.	−15.0 ± 0.3	−14.0 ± 0.1	−13.4 ± 0.2

All values are in kcal/mol and experiment data is from Ref. 27.

**Table 2:** The occupancy of the bridging water and the average distances of hydrogen bonds with the bridging water involved for APV and DRV bound HIV-1 PR complex systems.

	**WT-APV**	**Flap+-APV**	**Act-APV**	**WT-DRV**	**Flap+-DRV**	**Act-DRV**
Occupancy (%)	19.3 ± 4.8	17.2 ± 5.8	24.8 ± 3.9	99.3 ± 1.0	98.7 ± 5.3	26.1 ± 6.4
Average Distance (Å)	3.02 ± 0.38	3.01 ± 0.28	3.00 ± 0.36	2.92 ± 0.19	2.98 ± 0.22	3.01 ± 0.34
