# Supplementary material for: Effects of drug-resistant mutations on the dynamic properties of HIV-1 protease and inhibition by Amprenavir and Darunavir
Source: Sci Rep. 2015 May 27;5:10517. doi: 10.1038/srep10517 (PMC4444956; doi:10.1038/srep10517)
Supplement: Supplementary Information [file srep10517-s1.pdf]

Supplementary data

Effects of Drug-resistant Mutations on the Dynamic Properties of HIV-1 Protease and Inhibition by Amprenavir and Darunavir

Yuqi Yu,[1 §] Jinan Wang,[1 §] Qiang Shao,[1*] Jiye Shi,[2*] Weiliang Zhu[1*]

[1]Drug Discovery and Design Center, ACS Key Laboratory of Receptor Research, Shanghai Institute of Materia Medica, Chinese Academy of Sciences, 555 Zuchongzhi Road, Shanghai, 201203, China

[2]UCB Pharma, 216 Bath Road, Slough SL1 4EN, United Kingdom

[§]These authors contributed equally to the work.

*To whom correspondence should be addressed. Qiang Shao, Tel: +86 21 50806600-1304, E-mail: qshao@mail.shcnc.ac.cn; Jiye Shi, Jiye.Shi@ucb.com; Weiliang Zhu, wlzhu@mail.shcnc.ac.cn.

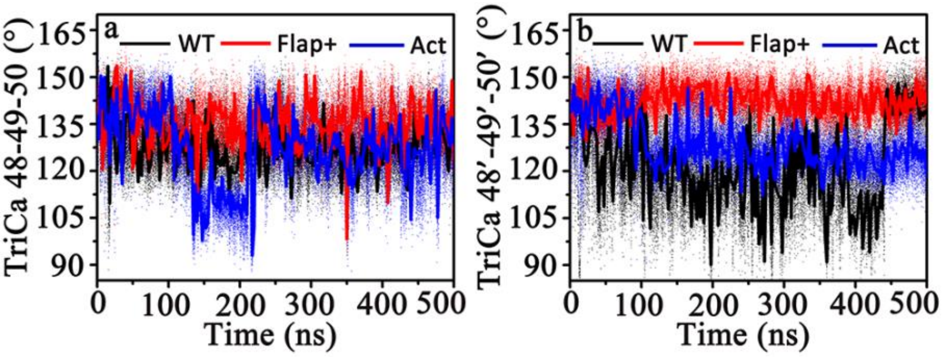

Figure S1. Time series of TriCa values of (a) G48-G49-I50 and (b) G48’-G49’-I50’ in WT HIV-1 PR and its Flap+ and Act variants.

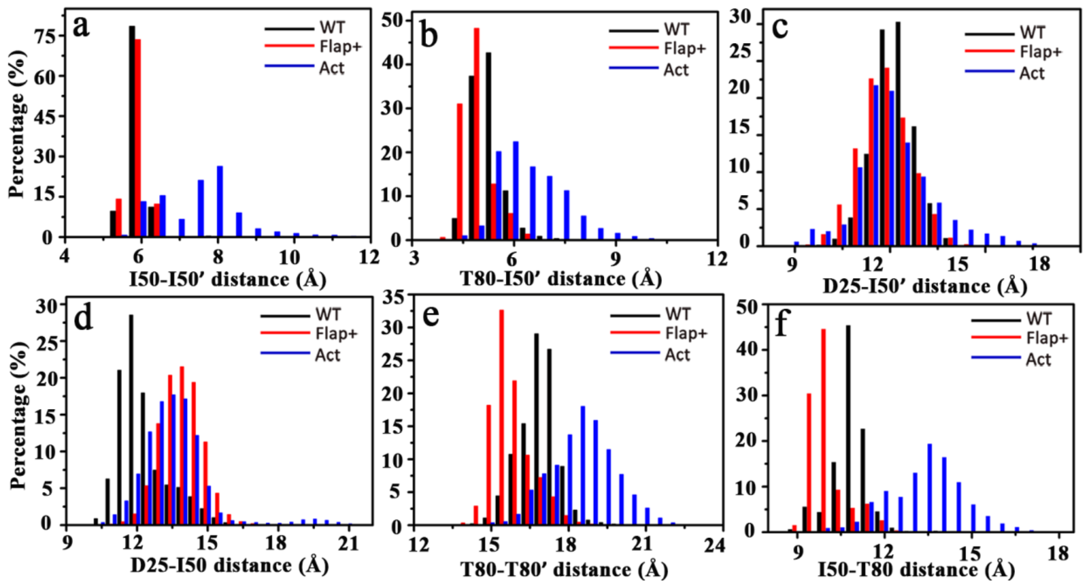

Figure S2. Distribution of (a) I50-I50’, (b) T80-I50’, (c) D25-I50’, (d) D25-I50, (e) T80-T80’, and (f) I50-T80 Cα atom distances of the apo WT HIV-1 PR and its Flap+ and Act variants.

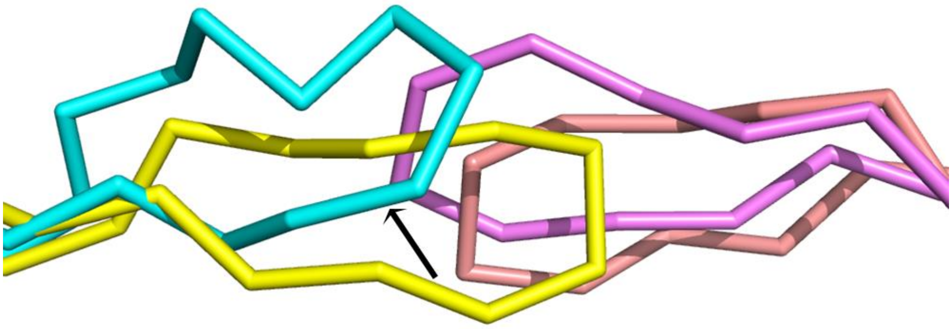

Figure S3. Comparison of a slightly upward motioned conformation of the flaps in Act variant observed in MD simulation to the closed conformation. The flaps in chains A and B in closed conformation are shown with pink and yellow ribbons and the corresponding flaps in upward motioned conformation are shown with purple and cyan ribbons. Direction of the motion of flap B is highlighted by black arrow.

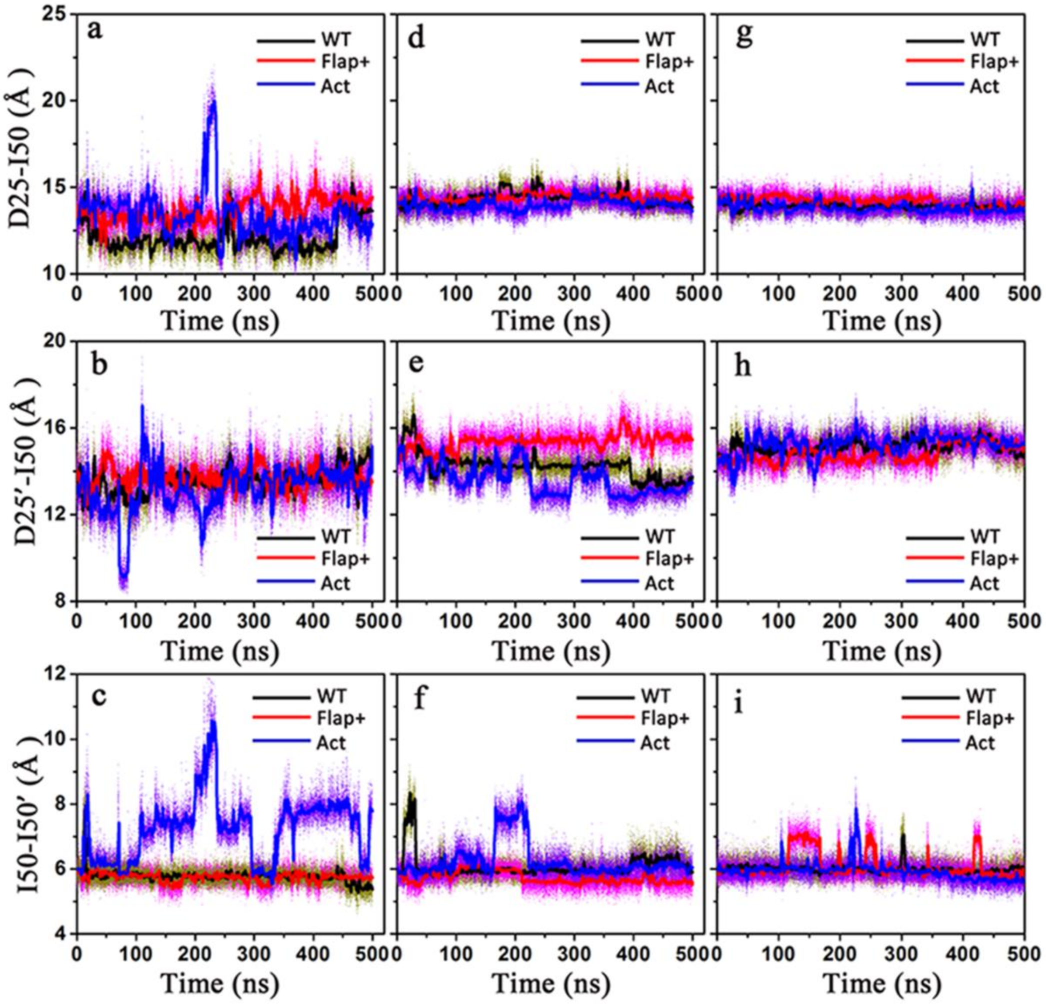

Figure S4. Time series of the Cα atom distances of D25-I50, D25’-I50’, and I50-I50’ for (a-c) apo, (d-f) APV-bound, and (g-i) DRV-bound WT HIV-1 PR and its Flap+ and Act variants, respectively.

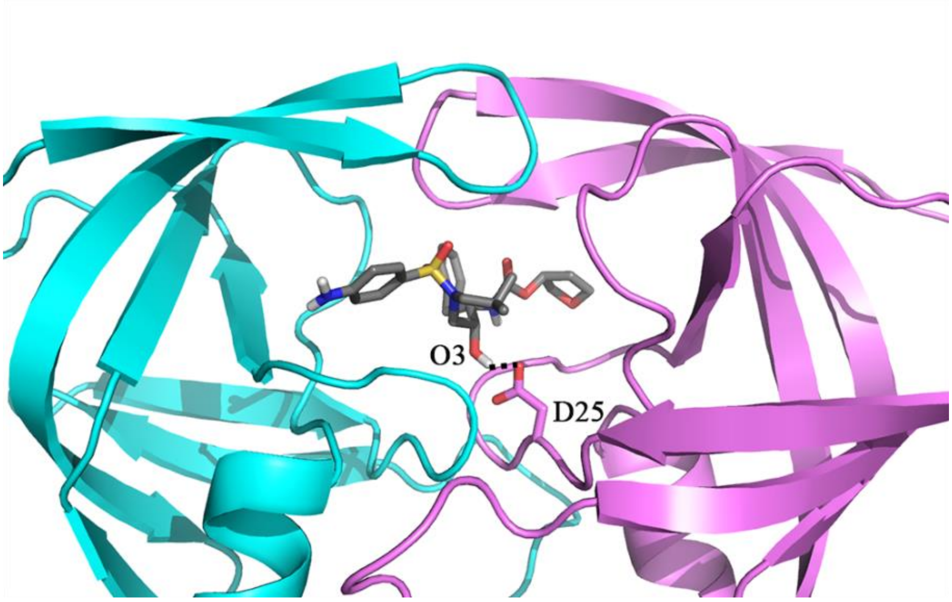

Figure S5. Hydrogen bond formed between the hydroxyl group of the inhibitor (APV or DRV) and the side-chain of D25 in HIV-1 PR. HIV-1 PR is showed with cartoon representation, the inhibitor and the side-chain of D25 are showed with licorice representation, respectively.

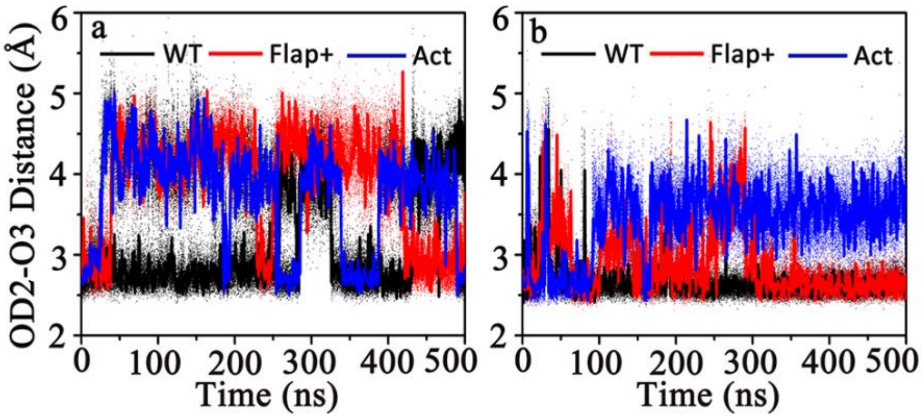

Figure S6. Time series of the distance between side-chain OD2 atom of D25 in HIV-1 PR and the hydroxyl oxygen (O3) of (a) APV or (b) DRV.

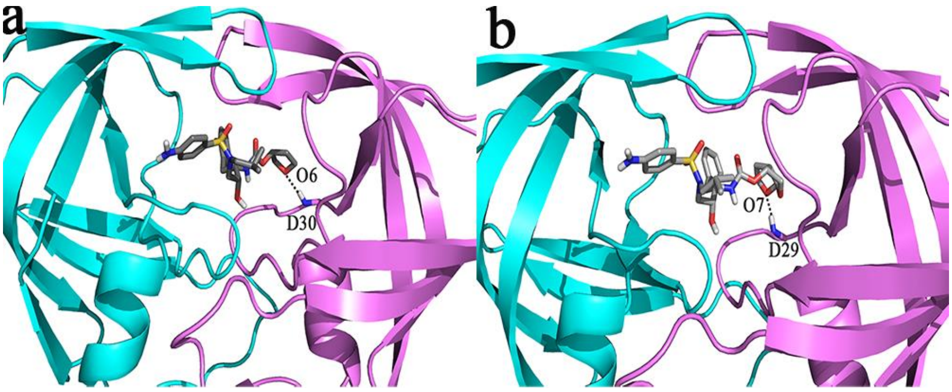

Figure S7. Hydrogen bonds formed between (a) the O6 oxygen of the inhibitor and D30, (b) the O7 oxygen of the inhibitor and D29 of HIV-1 PR. HIV-1 PR is showed with cartoon representation, the inhibitor and the backbone of D29 and D30 are showed with licorice representation, respectively.

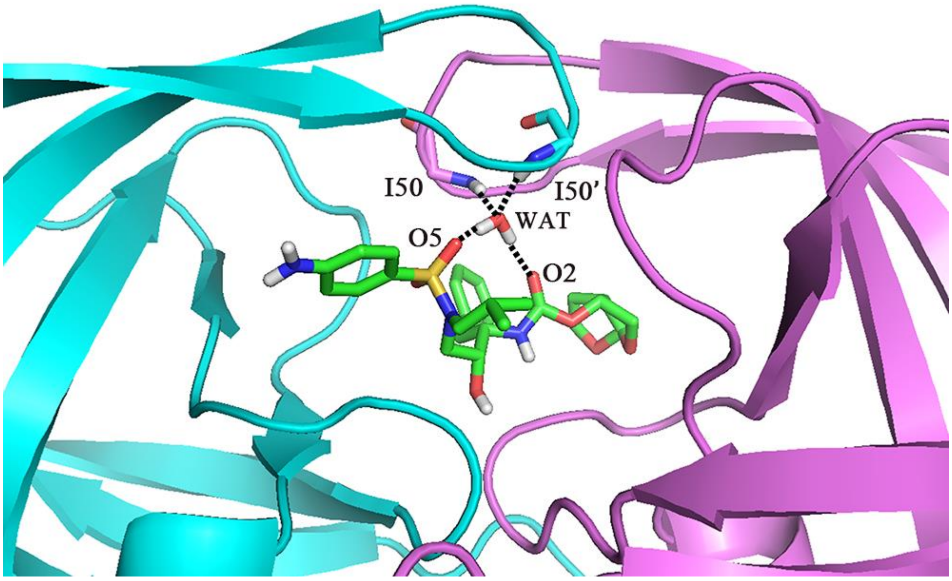

Figure S8. Hydrogen bonds formed among the bridging water molecule, the I50/I50’ in flap tips of HIV-1 PR, and APV/DRV inhibitor.
